# Molecular Mechanisms Behind the Role of Plasmacytoid Dendritic Cells in Systemic Sclerosis

**DOI:** 10.3390/biology12020285

**Published:** 2023-02-10

**Authors:** Inês S. Silva, Beatriz H. Ferreira, Catarina R. Almeida

**Affiliations:** 1 Institute of Biomedicine (iBiMED) and Department of Medical Sciences, University of Aveiro, 3810-193 Aveiro, Portugal; 2 Aveiro Institute of Materials (CICECO), Department of Chemistry, University of Aveiro, 3810-193 Aveiro, Portugal

**Keywords:** systemic sclerosis, plasmacytoid dendritic cells, fibrosis, autoimmunity, CXCL4, type I IFN, ER stress

## Abstract

**Simple Summary:**

Systemic Sclerosis (SSc) is a rare autoimmune disease characterized by scarring of the tissues and organs. This scarring, or fibrosis, has a major impact on a patient’s quality of life; for example, lung fibrosis reduces the efficiency of breathing and is a significant cause of death for SSc patients. To develop novel therapeutic strategies, it is important to understand what causes fibrosis to occur and progress. It was recently found that plasmacytoid dendritic cells (pDCs) are key immune cells in the development of fibrosis in SSc. These cells are innate immune cells that are specialized in anti-viral responses, and they are also involved in autoimmune diseases. This review will examine the recent research that has started to uncover the molecular mechanisms through which pDCs impact SSc.

**Abstract:**

Systemic sclerosis (SSc) is a debilitating autoimmune disease that affects multiple systems. It is characterized by immunological deregulation, functional and structural abnormalities of small blood vessels, and fibrosis of the skin, and, in some cases, internal organs. Fibrosis has a devastating impact on a patient’s life and lung fibrosis is associated with high morbimortality. Several immune populations contribute to the progression of SSc, and plasmacytoid dendritic cells (pDCs) have been identified as crucial mediators of fibrosis. Research on murine models of lung and skin fibrosis has shown that pDCs are essential in the development of fibrosis, and that removing pDCs improves fibrosis. pDCs are a subset of dendritic cells (DCs) that are specialized in anti-viral responses and are also involved in autoimmune diseases, such as SSc, systemic lupus erythematosus (SLE) and psoriasis, mostly due to their capacity to produce type I interferon (IFN). A type I IFN signature and high levels of CXCL4, both derived from pDCs, have been associated with poor prognosis in patients with SSc and are correlated with fibrosis. This review will examine the recent research on the molecular mechanisms through which pDCs impact SSc.

## 1. Systemic Sclerosis

Systemic sclerosis (SSc, also known as scleroderma) is a rare autoimmune disease that causes the progressive fibrosis of tissues and organs [1,2]. Despite its rarity, SSc is responsible for a significant number of deaths among all rheumatic disorders. Based on the extent of skin fibrosis, SSc can be classified into two types: limited cutaneous (lcSSc) or diffuse cutaneous (dcSSc), with the latter having more extensive skin involvement. These two types have different clinical associations and specific autoantibody profiles, with anti-centromere antibodies (ACA) being more prevalent in lcSSc, whereas anti-topoisomerase I (ATA) and anti-RNA polymerase III antibodies (ARAs) are more frequent in dcSSc [1,2]. In addition, there are patients who have no skin involvement but present clinical and serological features of SSc, and are classified as SSc *sine* scleroderma (also referred to as non-cutaneous SSc) [1].

Organ involvement progresses quickly in the early stages of the disease, highlighting the importance of early identification and intervention. Due to the significant patient-to-patient variability, a shift is needed in the classification of SSc diagnoses and treatments to focus on the molecular mechanisms rather than the clinical symptoms. It is recommended to test for SSc-specific-associated autoantibodies, which not only aid in diagnosing the cutaneous subtype but also provide information on clinical correlates [3].

Lung fibrosis is a common complication in SSc, and it is the principal cause of death related to the disease [4]. Other pathologies can also lead to lung fibrosis and death, such as idiopathic pulmonary fibrosis (IPF), the most common fibrosing interstitial lung disease (ILD). Although the outcome is similar, the mechanisms that lead to lung fibrosis are different. For that reason, it is important to study SSc-ILD and IPF separately and compare the findings to better understand the similarities and differences between the two pathologies, in order to develop more effective therapies. Both SSc-ILD and IPF involve the immune system, but the specifics of their involvement differ. The majority of SSc-ILD patients are positive for autoantibodies, with the presence of ATA and the absence of ACA increasing the likelihood of progressive ILD. In contrast, IPF patients do not have autoantibodies [5]. Despite these differences, both SSc-ILD and IPF lung tissues show an increase in genes related to fibrosis and insulin growth factor signaling [6]. Genes that are enriched only in SSc-ILD lungs are primarily associated with the regulation of vasculature development, immunity, and inflammation, while genes that are enriched only in IPF are mainly related to lymphocyte chemotaxis, emphasizing the role of external factors in the pathology of IPF [7].

### Systemic Sclerosis Etiology and Pathogenesis

The underlying cause of SSc is not known and its pathogenesis is complex and not fully understood. It is believed that the disease results from a combination of genetic factors and environmental influences. Specific polymorphisms in HLA genes have been linked to increased susceptibility to SSc [8,9,10]. Environmental factors that have yet to be identified may also play a role in triggering the development of SSc by an aberrant activation of the immune system or vascular damage [11].

SSc is characterized by three hallmarks: vasculopathy, immune dysregulation, and fibrosis. It is believed that the disease begins with vascular damage, and that it leads to the activation and recruitment of innate immune cells [1,2]. However, it has also been proposed that SSc is triggered by activated immune cells targeting endothelial cells of the blood vessels and causing vascular injury [11]. Regardless of the initial trigger, the recruitment of immune cells in response to vascular damage leads to chronic inflammation, increased vascular damage, and abnormal wound repair. The inflammatory environment also leads to the activation and dysregulation of fibroblasts, resulting in the excessive production of extracellular matrix (ECM) components and fibrosis. This fibrosis further promotes the recruitment and activation of immune cells, creating a feedback loop [1,2,11]. Simultaneously, the differentiation of myofibroblasts from different progenitors also exacerbates fibrosis [2]. Therefore, the current treatment strategies include the use of immunosuppressors and anti-fibrotic agents. Unfortunately, due to the complexity of the pathology and the difficulty in early diagnosis, these therapies are frequently ineffective, resulting in irreversible damage in the affected organs.

## 2. Immune Involvement in Systemic Sclerosis

Immune dysregulation in SSc is characterized by the activation and recruitment of the immune cells, such as macrophages, mast cells, plasmacytoid dendritic cells (pDCs), B cells, and T cells, from the blood and lymphoid organs into the skin, lungs, and other internal organs. Additionally, it is associated with the production of autoantibodies and cytokines, such as interleukin (IL)-1, IL-4, IL-6, and IL-13, transforming the growth factor (TGF)-β and C-X-C motif ligand 4 (CXCL4) [2,11]. The involvement of dysregulated immune responses in fibrosis has been recently reviewed elsewhere [12]. In this section, we will provide a brief overview of the contribution of adaptive and innate immunity to SSc and will focus on the role of dendritic cells (DCs).

### 2.1. Adaptive Immunity

In SSc, the balance between the regulatory and effector lymphocytes is disrupted. B cells are hyperactivated and have an impaired regulatory capacity [13], producing autoantibodies that are closely associated with the disease [2]. Peripheral B-cell homeostasis is thus disturbed, with patients exhibiting an expansion of naïve B cells and a reduction in memory B cells [14]. On the other hand, regulatory B cells (Bregs) are significantly decreased in SSc patients [15], and the depletion of these cells correlates with an increase in fibrosis severity [16]. Regarding the role of T cells, studies have reported an imbalance in the T helper type 17 (Th17)/regulatory T (Treg) cells ratio in the circulation of SSc patients [17,18,19]. Although both T-cell subtypes depend on TGF-β for their differentiation [17], the simultaneous presence of IL-6 favors Th17 differentiation in detriment of Treg [20], which explains the increased levels of Th17 cells in SSc patients’ blood [17,18] and skin [19]. IL-17A, which is produced by these cells, is also increased in these patients [21,22]. However, its role in the disease is controversial, since it has been implicated in both the increase [19] and decrease [22,23] of collagen production and deposition. Th2 cells are also linked with wound healing and fibrosis, and the CD4^+^ T cells in the skin of SSc patients exhibit a Th2-cell phenotype. It has been suggested that Th2 and secreted IL-4 are important players in the onset of fibrosis in SSc [24,25,26,27]. Moreover, IL-22-producing Th22 cells are also increased in SSc-ILD patients, and the serum IL-22 levels are positively correlated with ILD [28,29].

At the heart of SSc pathogenesis are the cytokines IL-6 and TGF-β. TGF-β is a profibrotic factor as it activates fibroblasts, stimulates collagen synthesis, and promotes myofibroblast differentiation [1,11]. Besides its direct role in fibrosis, TGF-β is also involved in Treg and Th17 differentiation [17], and the simultaneous presence of TGF-β and IL-6 skews Th17-cell differentiation [20]. IL-6 also promotes the polarization of T cells into Th2 cells [30]. Increased levels of IL-6, which can be found in the sera [24] and skin of SSc patients [31], correlate with disease severity, in particular with the extent of skin [1] and pulmonary involvement [5], and predict SSc-ILD decline and mortality [5]. IL-6 is in fact a crucial player in fibrosis, as both inhibiting IL-6 and blocking its receptor can improve fibrosis by suppressing fibroblast activation and differentiation [32].

### 2.2. Innate Immunity

Innate immunity is the first line of response of the immune system, and an important contributor to inflammation. Different innate cells, as well as intra- and extracellular players have been found to contribute to SSc. Nucleotide-binding and oligomerization domain (NOD)-like receptor protein 3 (NLRP3) is an intracellular pattern recognition receptor (PRR) that, together with an adaptor ASC (apoptosis-associated speck-like protein containing CARD), forms a complex known as inflammasome, which activates caspase 1. Fibroblasts from SSc patients have an activated inflammasome [33]. Interestingly, the inhibition or depletion of NLRP3 and caspase 1 reduces fibrosis both in in vitro and in vivo models [33]. The mechanism behind the contribution of NLRP3 inflammasome to fibrosis seems to involve the regulation of microRNA (miRNA), such as miR-155, whose expression correlates with the progression of lung fibrosis in SSc-ILD patients [34,35].

The role of macrophages has been reported for decades, with studies showing their strong activation as well as increased serum levels and skin infiltration in SSc patients [36,37,38]. Indeed, macrophage migration and infiltration correlate with disease severity and progression [39], while macrophage depletion or inhibition of its recruitment lead to amelioration in the skin and lung fibrosis in a *Jun*-inducible scleroderma murine model [40,41]. Macrophages can adopt different profiles, and in SSc, the skin and blood are enriched in alternatively activated (or M2-like) macrophages [37]. These are major sources of TGF-β, IL-10, cytokines with pro-fibrotic and anti-inflammatory properties, and are also involved in the recruitment of tissue-regenerating cells [42]. Chronic activation of M2--like macrophages in unresolved lesions leads to fibroblasts activation through the continuous production of growth factors and TGF-β, which exacerbates fibrosis instead of promoting controlled tissue regeneration [43].

Other innate immune cells have also been implicated in SSc pathogenesis, in particular, innate lymphoid cells-2 (ILC2) and mast cells, as both cell types infiltrate the skin of SSc patients. Furthermore, ILC2 [44,45] and mast-cell [46] activation have been correlated with fibrosis, in part due to the secretion of pro-fibrotic mediators [46,47]. Neutrophils are cells that are recruited in response to inflammation that have the capacity to activate fibroblasts through the production of reactive oxygen species (ROS) and pro-fibrotic cytokines, as TGF-β, IL-6, and vascular endothelial growth factor (VEGF) [48]. However, their activation state in SSc patients seems to depend on the stimulus they find, as others suggest that neutrophils from SSc patients have an impairment in functions such as cell migration, phagocytosis of bacteria, and neutrophil extracellular trap (NET) formation when stimulated with G-CSF [49,50]. More studies are necessary to clarify their role in SSc.

A cell population that has raised a particular interest are the dendritic cells (DCs), with studies reporting functional changes in DCs as well as altered tissue distribution, in both SSc patients and mice models of the disease [51].

### 2.3. Dendritic Cells

DCs are professional antigen-presenting cells that link the innate and adaptive immune system. They are specialized in pathogen-sensing and antigen presentation and are essential for the activation of naïve T cells. DCs express PRR, such as Toll-like receptors (TLRs), NOD-like receptors (NLRs), C-type lectin receptors (CLRs), and retinoic acid-inducible gene I (RIG)-I-like receptors (RLRs), to recognize pathogen- or danger-associated molecules in the extracellular and intracellular environment [51]. Additionally, they also constitutively express cGMP-AMP synthase (cGAS), which recognizes nucleic acids in the cytosol [52]. Upon recognition, DCs upregulate co-stimulatory signal molecules and secrete immune mediators that are required to regulate the response of T cells and other cell types [51].

Skin biopsies from SSc patients with a higher modified Rodnan skin score (mRSS) exhibited increased expression of DC signature genes, and the DCs displayed copious chromatin divergence between healthy and disease states [53]. ATAC-seq revealed that accessible DNA regions of skin-resident DCs are enriched for SSc-associated single-nucleotide polymorphisms (SNPs), which suggests that DCs are affected by genetic variants linked to SSc [53].

DCs are commonly characterized into three major populations: the conventional DC (cDC)1, cDC2, and the plasmacytoid DC (pDC) [54]. cDC1 is the primary cDC subset engaged in antigen cross-presentation and induction of antiviral T-cell responses, while cDC2 mainly induce T-cell responses against extracellular pathogens and allergens. pDCs are mostly involved in anti-viral immune responses and autoimmunity, due to their capacity to quickly secrete type I interferon (IFN). DCs can also be derived from monocytes upon stimulation, identified as monocyte-derived DCs (moDCs).

cDCs and moDCs obtained from SSc patients were shown to secrete high levels of IL-6 and tumor necrosis factor (TNF)-α upon TLR2, TLR3, and TLR4 stimulation, with TLR2 and TLR4 activation also inducing high quantities of IL-10 [55]. Additionally, cDCs and moDCs from SSc patients with a rare polymorphism in the *TLR2* gene showed an increased production of IL-6 and TNF-α upon TLR2 stimulation [56]. Furthermore, moDCs from SSc, both unstimulated and TLR-stimulated, produced high quantities of IL-33 [57]. In the same study, the co-culture of autologous T cells and TLR-stimulated moDCs from SSc patients promoted Th2, Th17, and dual Th2/Th17-cell phenotypes [57], showing that these DCs can shift the balance in Th responses. Therefore, these data show that different DC populations are involved in the pathogenesis of SSc. Still, pDCs are the subtype that appears to be more relevant for SSc pathogenesis, in particular, for the development of fibrosis.

## 3. Plasmacytoid Dendritic Cells

pDCs are innate immune cells that reside in and recirculate through lymphoid organs, constituting 0.1–0.5% of human peripheral blood mononuclear cells (PBMCs) [58,59]. These cells play an important role in the body’s initial response to viral infections by producing large amounts of type I IFN. They have also been linked to the development of autoimmune diseases such as systemic lupus erythematosus (SLE), psoriasis, and SSc [59]. Human pDCs express clusters of differentiation (CD)4, CD45RA, CD68, immunoglobulin-like transcript (ILT)3, and CD123 (IL-3 receptor α-subunit), and can be identified by a set of markers, including blood dendritic cell antigen (BDCA)2 (CD303), BDCA4 (CD304), and ILT7 [59,60]. Among these markers, BDCA2 is the most specific and sensitive for identifying pDCs in the tissues, since BDCA4 and CD123 can also be expressed in non-hematopoietic and neoplastic cells. pDCs have the morphological appearance of antibody-secreting cells, hence the plasmacytoid designation, and were originally defined as “natural IFN-producing cells”.

The activation of pDCs through TLR7 and TLR9 induces the secretion of pro-inflammatory cytokines and chemokines [59]. This response is triggered by the recognition of single-stranded RNA (ssRNA) and unmethylated CpG motif-containing DNA, respectively, and leads to the secretion of high quantities of type I IFN, as early as 1–3 h after activation [58]. The production of type I IFN by pDCs can be up to 1.000 times more potent than in other cells. This initiates an antiviral response in adjacent cells, and also leads to the activation and migration of natural killer (NK) cells, the maturation of other DCs and macrophages, the T-cell response, and the differentiation of plasma cells [58,60]. Besides type I IFN, pDCs can also secrete other pro-inflammatory cytokines and chemokines, including IL-6, IL-12, CXCL8, CXCL10, chemokine (C-C motif) ligand (CCL)3, CCL4, and TNF-α [60]. Some studies have also suggested that pDCs can present antigens and prime T cells, but this remains a controversial topic [59,61].

The origin of pDCs remains a subject of debate in the scientific community [62,63]. Early studies suggested that pDCs can develop in the bone marrow (BM) through myeloid and lymphoid pathways, from common DC progenitors (CDPs) or common lymphoid progenitors (CLPs), respectively [58]. However, more recent transcriptional and functional studies in mice have shown that pDCs generated from these different pathways may have distinct properties. pDCs derived from myeloid origin do not produce type I IFN in response to TLR9 stimulation, and have an antigen presentation capacity, being more similar to cDCs, and thus classified as pDC-like cells [64]. Others have suggested that pDCs and B cells have a shared origin [65], but recent studies, which were also performed in mice, found no common progenitor for pDCs and B cells [64]. Ziegler-Heitbrock et al. [62] recently discussed the properties of pDCs from myeloid and lymphoid origin, and proposed that pDCs should be classified as innate lymphocytes rather than DCs due to their similarities with innate lymphocytes.

As referred to above, the capacity of pDCs for antigen presentation in addition to secreting type I IFN has been a topic of debate for several years. Some authors have questioned whether the description of antigen presentation in bulk pDC preparations is due to cell contamination, such as the presence of Axl^+^ DC, a pDC-like cell lineage that is inefficient at producing type I IFN but can efficiently activate T and B cells [66,67]. Another possibility is that different functions are associated with specific pDC subsets. Earlier studies have found that pDC subsets can be distinguished based on CD2 expression [68,69,70]. Later, it was described that, both in humans and mice, pDCs expressing CD2^hi^, CD5, and CD81 have a reduced capacity to produce type I IFN, but show the ability to stimulate B- and T-cell activation. On the other hand, CD5^-^CD81^-^ pDCs do not stimulate B or T cells, but they produce high amounts of type I IFN [71]. Other studies have described the emergence of three distinct subsets of pDCs when they are stimulated: PD-L1+CD80– (P1), PD-L1+CD80+ (P2), and PD-L1–CD80+ (P3). These subsets seem endowed with a different functional role, with P1 cells specializing in the production of type I IFN, P3 in activation of adaptive immunity, and P2 performing both functions [72]. More recently, a study followed individual pDCs upon infecting mice with cytomegalovirus (CMV), and found that type I IFN production and T-cell activation could be performed by the same cell, but in a sequential manner [61]. Therefore, it was suggested that pDCs have a functional plasticity, in a way that upon viral stimulation, cells will first produce anti-viral type I IFN, and later, they will be capable of antigen presentation and activation of T cells [61].

## 4. Plasmacytoid Dendritic Cells in Systemic Sclerosis

### 4.1. Relevance of pDCs in SSc Pathogenesis

In healthy individuals, pDCs are mostly found circulating between the lymphoid organs and they only migrate to tissues upon inflammation [60]. However, in patients with SSc, pDCs are mainly found in the skin and lungs, and their levels in the blood are decreased [73]. Moreover, the severity of lung disease in SSc patients is correlated with the frequency of pDCs found in the lungs [73]. Importantly, pDCs play a direct role in causing and maintaining fibrosis, as their depletion has been shown to improve skin and lung fibrosis, as described in Table 1.

The presence of pDCs in the lungs appears to be a feature of pulmonary fibrosis, since their frequency in the lungs is similar in both SSc-ILD and IPF patients [74]. However, the mechanisms by which they contribute to lung fibrosis might differ between diseases, as pDCs in SSc-ILD are more transcriptionally active, have multiple cellular stress-related pathways upregulated, and have an increased expression of type I IFN receptor (IFNAR) [74].

**Table 1:** **Impact of pDC Depletion in Fibrosis.** Summary of studies where pDCs have either been depleted or inhibited, and the effect in fibrosis was measured. Downward arrows (↓) indicate a decrease.

Treatment of pDC Depletion/Inhibition	Model	Impact in Fibrosis	Reference
In vivo depletion of pDCs by injection ^1^ with anti-PDCA-1	Bleomycin-induced fibrosis in C57BL/6 mice	↓ clinical severity score; ↓ histopathological changes in lungs; ↓ skin fibrosis; and ↓ total collagen and fibrillar collagen in skin and lung fibrosis	[73]
Imatinib ^2^ treatment	Systemic sclerosis patients	↓ pDCs in bronchoalveolar lavage, but not in peripheral blood; improved/stable SSc-ILD (note that pDCs were already reduced in peripheral blood of these patients)	[73]
Diphtheria toxin injection starting 24 h before bleomycin injection	Bleomycin-induced fibrosis in CLEC4C-DTR ^3^ mice	↓ pDCs infiltration in the skin; ↓ of skin thickness; ↓ α-SMA positive dermal myofibroblasts; and ↓ TGF-β expression	[75]
BDCA-2 targeting using CBS004	Bleomycin-induced fibrosis in NOD SCID mice injected with purified human primary pDCs and treated with topical 5% imiquimod	Retention of fatty layer tissue; ↓ of dermal and epidermal thicknesses; ↓ of collagen content; ↓ and of MX1 and specific pSTAT1 dermal fibroblast protein expression	[76]

^1^ A total of 48 h before and 14 days after the first bleomycin injection. ^2^ Tyrosine kinase inhibitor that targets enzymes involved in fibrotic pathways. ^3^ Transgenic mice that express diphtheria toxin receptor under the control of CLEC4C, a highly specific human pDC promoter. Administration of diphtheria toxin selectively ablates pDC.

### 4.2. pDCs Secrete CXCL4

pDCs from SSc patients secrete CXCL4 and, upon activation, IFN-α [75], which creates an inflammatory environment in the tissues that they infiltrate. Increased levels of CXCL4 are found in the blood and skin of SSc patients [77], and they correlate with the presence [77] and progression [78] of complications, such as ILD and pulmonary hypertension (PH). Furthermore, both blocking CXCL4 and deleting the *CXCL4* gene reduce skin and lung fibrosis in a mouse model of the disease using bleomycin [79], which confirms the role of CXCL4 in disease pathogenesis.

pDCs from SSc patients aberrantly express TLR8, which is not expressed in healthy conditions [75]. This abnormal expression contributes to disease progression, since signaling through TLR8 induces the production of CXCL4 [75]. Additionally, TLR8 expression leads to an increased infiltration of pDCs into the tissues, exacerbating the disease and resulting in worse skin fibrosis [75]. TLR9 activation in the presence of hypoxia was also shown to induce CXCL4 production [80].

CXCL4 can form complexes with self or foreign DNA, and these complexes can then bind and activate pDCs through TLR9, independent of the CXCL4 receptor CXCR3 [81]. Anti-CXCL4 antibodies can exacerbate this by binding to the stimulatory CXCL4-DNA complexes and inadvertently increasing their activity [82]. By amplifying TLR9-induced pDC activation, CXCL4 potentiates IFN-α production [75,81,82], contributing to the type I IFN signature seen in SSc patients [83,84]. Although CXCL4 appears to be more effective and relevant, other chemokines such as CXCL10, CXCL12, and CCL5 can also complex with DNA and induce TLR9-mediated type I IFN production [85]. Additionally, CXCL4 was recently found to complex with self RNA, leading to pDC activation through TLR7/8 [86,87]. These CXCL4-RNA complexes not only correlate with the type I IFN signature, but also with TNF-α levels in SSc plasma [87].

**Figure 1:**
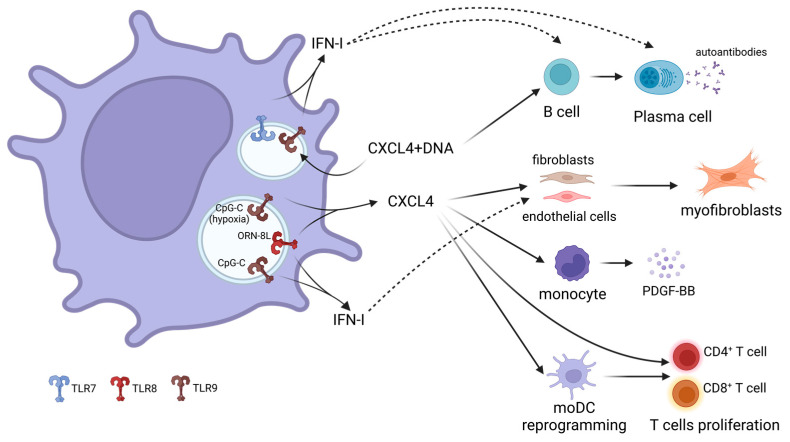
**The critical role of CXCL4 in the pathogenesis of SSc.** CXCL4 is aberrantly secreted by pDCs in SSc [77]. It is produced upon TLR9 stimulation under hypoxic conditions [80] or upon TLR8 activation [75]. CXCL4 forms complexes with DNA, which stimulate TLR9-induced IFN-α production by pDCs [81], creating a harmful feedback loop that exacerbates pDCs innate capacity to produce high quantities of type I IFN [81,82]. Additionally, CXCL4 directly promotes differentiation of different cells into myofibroblasts, the main profibrotic cell in SSc [79]; increases the secretion of PDGF-BB by monocytes, driving fibroblasts activation [88]; alters moDC differentiation, maturation and activity [89,90]; induces T-cell proliferation [82,90]; and, by forming complexes with DNA, stimulates B cells and induces them to produce anti-CXCL4 antibodies, which can bind to CXCL4-DNA complexes and increase their activity, contributing to disease progression [82]. IFN-I: type I IFN. Created with https://biorender.com/ (accessed on 3 February 2023).

CXCL4 plays a central role in a feedback loop that contributes to increased inflammation and fibrosis [81] (Figure 1). It can directly promote the differentiation of different cells into myofibroblasts, which produce collagen and other ECM components, contributing to fibrosis [79]. Furthermore, CXCL4 stimulates B cells to secrete autoantibodies [82], induces the proliferation of SSc-derived T cells [82], and enhances the secretion of platelet-derived growth factor (PDGF)-BB by monocytes, a growth factor that is increased in dcSSc patients and has been shown to enhance fibronectin and collagen deposition by fibroblasts [88]. CXCL4 may also reprogram immune cells, as shown by the different transcriptomic and epigenetic profiles of moDCs differentiated in the presence of CXCL4. These cells exhibited drastic changes in genes related to metabolism and transcription, and upon stimulation with Poly(I:C), they showed an upregulation of the pathways involved in cellular adhesion, integrin signaling, ECM organization, and collagen formation [89]. These CXCL4-moDCs were also shown to induce CD4^+^ T-cell and CD8^+^ T-cell responses in an antigen-independent manner [90]. Overall, CXCL4 links immune dysregulation and fibrosis in SSc, a crucial step in disease progression. Thus, disrupting CXCL4 production or activity might be a promising target to stop disease progression and fibrosis onset.

### 4.3. The Impact of Type I IFN in SSc

Type I IFN is an important mediator of innate and adaptive immunity, being particularly relevant for antiviral responses. Moreover, type I IFN has been implicated in the pathogenesis of various rheumatic diseases, including SSc. A significant proportion of SSc patients (43–70%) [84,91,92] have been described as presenting a type I IFN signature [93], which is higher in SLE (73–93%) [91,94,95,96] and lower in rheumatoid arthritis (20–52%) [91,94,97]. Type I IFN can be produced by almost every cell in the body, but there is evidence of an IFN signature in monocytes at an early stage SSc [84]. Nevertheless, pDCs have been identified as one of the main producers of IFN-α in SSc, at least partially upon activation by CXCL4-DNA complexes, as described above. Indeed, the depletion of pDCs leads to an improvement in fibrosis and reverts the upregulation of IFN-regulated genes in a bleomycin-induced SSc model [75].

The exact role of type I IFN in the pathogenesis of SSc was initially unclear, with some studies suggesting an anti-fibrotic effect [98,99], while others showed evidence of a detrimental effect [92,100]. In fact, there are clinical cases of patients treated with type I IFN for certain pathologies, namely leukemia, hepatitis C, and multiple sclerosis, who develop SSc [101,102,103,104]. Additionally, polymorphisms in the genes of the type I IFN pathway, such as the IFN regulatory factor (IRF) 4, 5, 7, and 8, and the signal transducer and activator of transcription (STAT)4 [105,106,107,108], have been associated with SSc. Both IRF5 and STAT4 depletion were observed to improve fibrosis in bleomycin-induced SSc mice [109,110]. Moreover, IRF7 was found to be upregulated in PBMCs [111] and the fibrotic skin of SSc patients [112], and to complex with Smad3, a key component of the TGF-β signaling pathway. The finding that IFN-α induces collagen and fibronectin production by dermal fibroblasts and that IRF7 knock-out (KO) decreased this production [112] suggests that IFN-α has a role in fibrosis progression through IRF7. As a consequence, blocking IFNAR has been explored as a possible therapy for SSc [92,113,114].

Overall, the evidence of a correlation between genetic polymorphisms and disease susceptibility, combined with the newly discovered link between type I IFN signaling and TGF-β signaling pathways, points to type I IFN having an important role in SSc. Therefore, not only the inhibition of its activity through IFNAR blockage, but also the disruption of its production by pDCs may be a good therapeutic option for SSc patients.

### 4.4. Dysregulation of pDCs

The mechanisms behind the chronic activation and aberrant features of pDCs in SSc are not fully understood. Still, recent studies have provided some insight. For example, Rossato et al. [115] reported increased miR-618 expression in pDCs from SSc patients, even in the absence of skin fibrosis, and showed that this expression directly correlated with the presence of ILD [115]. Since it targets IRF8, a critical transcription factor for pDC development and activation, miR-618 overexpression suppresses pDC development while promoting IFN-α secretion [115]. IRF8 downregulation also appears to have a critical role in other immune cells that affect SSc pathogenesis, since it was also reported in monocytes from SSc patients and was shown to lead to M2-like macrophages [116]. Similarly, miR-126 and miR-139-5p are upregulated in circulating pDCs of SSc patients and correlate with the expression of IFN-stimulated genes (ISG) [117]. Downregulation of the Runt-related transcription factor 3 (RUNX3), a transcription factor involved in the differentiation of DC lineage, has also been described in pDCs from SSc patients and has been associated with altered pDC tissue distribution and increased maturation markers upon activation [118]. The downregulation of RUNX3 has been associated with *RUNX3* hypermethylation and hypoxia. The deletion of *RUNX3* spontaneously induced skin inflammation and fibrosis in wild-type (WT) mice and increased the severity of skin fibrosis in a bleomycin-induced model [118]. Although the knowledge on the molecular alterations that occur in pDCs in SSc is still limited, it appears that they have an important role in SSc. More research is needed to fully understand the extent of pDC dysregulation and its role in SSc pathogenesis.

### 4.5. The Role of Endoplasmic Reticulum Stress

Recently, it has been suggested that dysregulation of the response to endoplasmic reticulum (ER) stress by pDCs has implications in SSc pathogenesis [119]. ER stress occurs when there is an accumulation of unfolded or misfolded proteins, activating ER stress sensors (protein kinase R-like endoplasmic reticulum kinase (PERK), inositol-requiring kinase 1 (IRE1), and activating transcription factor 6 (ATF6)), and initiating the unfolded protein response (UPR). ER stress can be caused by a variety of triggers, including exposure to environmental particles, which is also a risk factor for developing lung fibrosis. Moreover, in conditions of high demand for protein synthesis, as during immune activation, the folding capacity of the ER might be exceeded, resulting in an accumulation of unfolded proteins.

ER stress has been implicated in SSc pathogenesis as it promotes apoptosis and inflammation [120]. The UPR favors cell proteostasis; however, if it fails and ER stress persists, it can activate cell death pathways. Studies have shown that mice deficient in the C/EBP homologous protein (CHOP), a promoter of ER stress-induced cell death, are protected from lung fibrosis induced by bleomycin, due to the reduced infiltration of M2-like macrophages in the lungs and the decreased TGF-β production [121]. Others also showed that the attenuation of ER stress suppressed the production of ECM by TGF-β-treated fibroblasts in vitro, and reduced lung fibrosis and inflammation in a bleomycin-induced fibrosis mice model [122,123]. In skin and lung fibroblasts derived from SSc patients, IRE1α inhibition/depletion abrogated the TGF-β-induced production of fibrotic proteins [124]. It was also observed that IRE1α can cleave certain miRNAs, including miR-150, which is involved in the inhibition of α-smooth muscle actin (α-SMA) expression [124]. Furthermore, the polarization of Th17 cells, which are involved in SSc pathogenesis, is promoted by ER stress induced by hypoxia or nutrient deprivation [125]. Together, these data suggest that cellular responses to ER stress may contribute to the progression of SSc.

**Figure 2:**
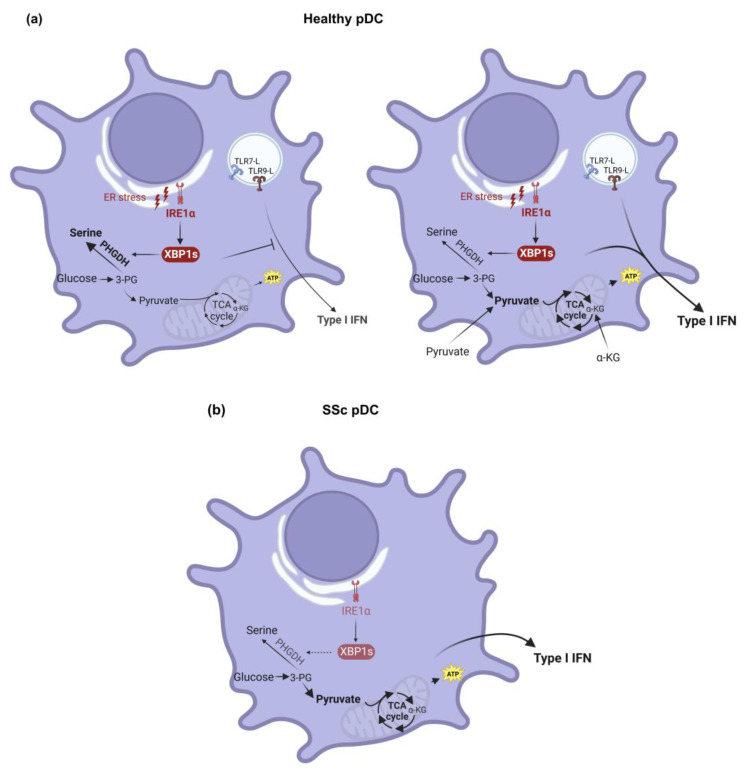
**The role of ER stress in type I IFN production by human pDCs.** (**a**) pDCs that are isolated from the blood of healthy donors (HDs) facing ER stress and TLR7/9 activation present an increase in the IRE1α-XBP1 pathway. In these conditions, there is upregulation of the PHGDH levels, favoring serine synthesis besides TCA cycle, reducing ATP levels, and leading to a decrease in the type I IFN production triggered by TLR activation (left). In the presence of pyruvate, the TCA cycle is potentiated, resulting in a synergy of ER stress and TLR7/9 activation and, thus, the production of high levels of type I IFN (right). (**b**) On the other hand, pDCs that are isolated from the blood of systemic sclerosis (SSc) patients present decreased levels of the IRE1α-XBP1 pathway. Thus, PHGDH levels are reduced compared with HDs, resulting in a pyruvate and α-ketoglutarate-mediated induction of the TCA cycle that promotes type I IFN production, contributing to the type I IFN signature observed in SSc [119]. Created with https://biorender.com/ (accessed on 3 February 2023).

ER stress may contribute to immune dysregulation, a hallmark of SSc. In DCs, TLR4 stimulation relies on PERK and eukaryotic initiation factor-2α (eIF2α) phosphorylation to amplify type I IFN expression [126]. As for pDCs, they have a large ER and rely heavily on the UPR to maintain proteostasis. Studies have shown that pDCs have increased basal levels of the IRE1α-regulated transcriptional factor X-box binding protein (XBP)1, which is essential for pDC development and survival [127]. Additionally, ER stress enhances IFN-β production by pDCs upon TLR7/9 stimulation in BM-derived pDCs [128], and in human pDCs when the culture media are supplemented with pyruvate [119] (Figure 2a). Interestingly, PBMCs from SSc patients were found to have an upregulation of UPR genes [129]. Moreover, single-cell analysis suggested an upregulation of the stress pathways in pDCs within the fibrotic lungs of SSc patients, compared to patients with IPF, where a role for pDCs has not been described [74]. However, the upregulated pathways were not identified. A more recent study showed that pDCs obtained from the blood of SSc patients have the IRE1α-XBP1 signaling pathway downregulated, which contributes to the type I IFN signature observed in this pathology [119] (Figure 2b).

ER stress has been shown to play a role in metabolic reprogramming, and IRE1α has been suggested to be involved in maintaining metabolic homeostasis [130]. It was found that the tricarboxylic acid (TCA) cycle, which is normally highly active in DCs [131], is required for optimal type I IFN responses in TLR9-activated pDCs [119]. In vitro experiments with pDCs from healthy donors (HDs), in the absence of pyruvate, showed that TLR-induced type I IFN production is inhibited by the IRE1α-XBP1–phosphoglycerate dehydrogenase (PHGDH) axis, which correlates with decreased ATP levels [119] (Figure 2a). In contrast, the downregulation of the IRE1α-XBP1 axis found in pDCs from SSc patients, which is associated with a decreased PHGDH expression, leads to the potentiation of the TCA cycle, and promotes type I IFN production even in the absence of TLR activation [119] (Figure 2b). This finding is consistent with the reported high levels of succinate and fumarate in the serum of SSc patients [132]. Altogether, these data suggest that ER stress is an important player in the pathogenesis of SSc, and that both ER stress modulation and metabolic reprograming of pDCs might be attractive therapeutic strategies for this disease.

## 5. Targeting pDCs as a Therapeutic Strategy

A search for “plasmacytoid dendritic cells” on clinicaltrials.gov does not reveal any ongoing trials specifically targeting pDCs for the treatment of SSc. However, because of the important role of these cells in other immune-mediated and rheumatic diseases, some pDC-targeting therapies are currently under investigation. If these drugs are found to be safe and effective, they may hold promise for the future treatment options for SSc. BIIB059, a humanized anti-BDCA2 monoclonal antibody, was shown in phase II trials to ameliorate skin disease in SLE and is currently in phase III [NCT05352919; https://clinicaltrials.gov (accessed on 24 January 2023)]. Daxdilimab (or VIB7734), an ILT7 blocker, is currently in phase II in SLE [(NCT04925934 and NCT05430854; https://clinicaltrials.gov/ (accessed on 24 January 2023)] and alopecia areata [NCT05368103; https://clinicaltrials.gov (accessed on 24 January 2023)]. Additionally, VIB1116, a new DC-targeting drug, is now undergoing phase I in patients with DC-mediated rheumatic diseases [NCT04948099; https://clinicaltrials.gov (accessed on 24 January 2023)]. Additionally, there are ongoing clinical trials that target IFNAR in SLE [phase III, NCT04877691; https://clinicaltrials.gov (accessed on 24 January 2023)], lupus nephritis [phase III, NCT05138133; https://clinicaltrials.gov (accessed on 24 January 2023)], and Sjogren’s syndrome [phase II, NCT05383677; https://clinicaltrials.gov (accessed on 24 January 2023)].

ER stress responses and metabolic changes in pDCs appear to contribute to the development of SSc. There a few clinical trials investigating the use of ER stress and metabolic modulators in different autoimmune conditions. In particular, tauroursodeoxycholic acid (TUDCA), an ER stress inhibitor, is being explored as a treatment for ulcerative colitis [phase I, NCT04114292; https://clinicaltrials.gov (accessed on 25 January 2023)]. Furthermore, a clinical trial with metformin, which decreases TCA cycle activity, is currently recruiting, to explore its effect on pulmonary hypertension (PH), an SSc complication [NCT01884051; https://clinicaltrials.gov (accessed on 25 January 2023)]. CXCL4 is also described as an important mediator on SSc, but we did not find any clinical trial specifically targeting this molecule. In the future, it will be interesting to follow the results of these clinical trials, as they might reveal promising therapies for the treatment of SSc.

## 6. Conclusions

SSc is a multi-organ complex disease that involves the interplay of several immune cells acting in concert in different tissues. Recently, it has become clear that pDCs, which are a rare cell population, are central in SSc pathology. Indeed, animal studies suggest that inhibiting pDCs might prevent or even ameliorate fibrosis. Different studies have addressed the molecular mechanisms that regulate pDC behavior in the context of SSc, and point to the importance of CXCL4, type I IFN, miRNA regulators, ER stress, and metabolic regulation.

In the future, it will be important to determine which specific pDC subsets are activated or recruited, and what are the underlying molecular pathways and triggers. Advances in single-cell technologies promise new opportunities for the in situ and ex vivo analysis of this rare cell population. A deeper understanding of the regulatory pathways that are altered in pDCs in SSc will contribute to defining novel therapeutic targets for halting, slowing, or altering the progression of this pathology.

## Data Availability

Not applicable.
